# Hydropriming Applied on Fast Germinating *Solanum villosum* Miller Seeds: Impact on Pre-germinative Metabolism

**DOI:** 10.3389/fpls.2021.639336

**Published:** 2021-03-25

**Authors:** Chiara Forti, Valentino Ottobrino, Enrico Doria, Laura Bassolino, Laura Toppino, Giuseppe Leonardo Rotino, Andrea Pagano, Anca Macovei, Alma Balestrazzi

**Affiliations:** ^1^Department of Biology and Biotechnology “Lazzaro Spallanzani”, University of Pavia, Pavia, Italy; ^2^CREA, Research Centre for Genomics and Bioinformatics, Montanaso Lombardo, Italy; ^3^CREA, Research Centre for Cereal and Industrial Crops, Bologna, Italy

**Keywords:** crop wild relative, *Solanum villosum*, hydropriming, pre-germinative metabolism, reactive oxygen species, antioxidant response

## Abstract

Seed priming can circumvent poor germination rate and uniformity, frequently reported in eggplant (*Solanum melongena* L.) and its crop wild relatives (CWRs). However, there is still a gap of knowledge on how these treatments impact the pre-germinative metabolism in a genotype- and/or species-dependent manner. The CWR *Solanum villosum* Miller (hairy nightshade) investigated in this study showed a quite unique profile of fast germination. Although this accelerated germination profile would not apparently require further improvement, we wanted to test whether priming would still be able to impact the pre-germinative metabolism, eventually disclosing the predominant contribution of specific antioxidant components. Hydropriming followed by dry-back resulted in synchronized germination, as revealed by the lowest *MGR* (Mean Germination Rate) and *U* (Uncertainty) values, compared to unprimed seeds. No significant changes in ROS (reactive oxygen species) were observed throughout the treatment. Increased tocopherols levels were detected at 2 h of hydropriming whereas, overall, a low lipid peroxidation was evidenced by the malondialdehyde (MDA) assay. Hydropriming resulted in enhanced accumulation of the naturally occurring antioxidant phenolic compounds chlorogenic acid and iso-orientin, found in the dry seeds and *ex novo* accumulation of rutin. The dynamic changes of the pre-germinative metabolism induced by hydropriming are discussed in view of future applications that might boost the use of eggplant CWRs for breeding, upon upgrade mediated by seed technology.

## Introduction

The limited genetic diversity of cultivated eggplant (*Solanum melongena* L.) is in contrast with the huge gene pool found in wild relatives (Meyer et al., 2012; Taher et al., 2017), a promising source of high-quality traits (Knapp et al., 2013; Rotino et al., 2014). Improved eggplant varieties will contribute to sustainable production under changing climate, since eggplant requires a long growth period during which it is exposed to pathogens, pests, and weeds. Reports describing the use of Crop Wild Relatives (CWRs) and allied species in eggplant breeding are only increasing in the last years (Rotino et al., 2014; Liu et al., 2015; Plazas et al., 2016). Production of healthy seedlings is often compromised by uneven ripening of seeds and fruits, resulting in low quality seeds (Carvalho Martins et al., 2012). Seed vigor and germination profiles are among the standardized descriptors used to recommend genotypes for breeding (Taher et al., 2017). Dormancy, low seed germination rate and uniformity, documented in eggplant CWRs (Demir et al., 1994; Adebola and Afolayan, 2006; Taab and Andersson, 2009), strongly delay or prevent their exploitation in breeding programs or their use as rootstocks. Low-cost, pre-sowing treatments (“seed priming”) can be used to improve germination. These techniques allow to carry out imbibition under controlled conditions, boosting the antioxidant and DNA repair responses when the pregerminative metabolism starts, while avoiding radicle protrusion and loss of desiccation tolerance (Heydecker et al., 1973; Burgass and Powell, 1984; Ashraf and Bray, 1993; Bailly et al., 1998; Paparella et al., 2015). Enhanced crop yields resulting from primed seeds are due to increased tolerance to biotic/abiotic stresses and individual plant performance. Due to these benefits, priming is gaining momentum as a strategy to address the current and future issues of sustainable crop production in adverse environments (Ibrahim, 2016; Wojtyla et al., 2016; Farooq et al., 2017; Macovei et al., 2017).

The empirical features of current priming protocols and the genotype- and seed lot-dependent variability delay the work of seed technologists, breeders, and seed bank operators. Molecular hallmarks (genes, proteins, and metabolites) are required to predict and/or monitor the effectiveness of novel pre-sowing treatments. Several reports have already highlighted the positive correlation between the antioxidant response and increased seed vigor (Gallardo et al., 2001; Bailly, 2004) and the impact of effective DNA repair on seed quality (Waterworth et al., 2019). A progressive increase in oxidative DNA damage occurring during seed imbibition was measured in terms of 7,8-dihydro-8-oxoguanine (8-oxodG) levels, in the model legume *Medicago truncatula* (Balestrazzi et al., 2011). The concomitant up-regulation of key BER (base excision repair) genes including OGG1 (8-oxoguanine glycosylase/lyase) and Tdp1a (tyrosyl-DNA phosphodiesterase) was also described (Macovei et al., 2010, 2011; Balestrazzi et al., 2011; Pagano et al., 2017, 2019; Forti et al., 2020a,b).

To date, there are reports describing the benefits and drawbacks of this technology applied to eggplant (Demir et al., 1994; Gomes et al., 2012; Gonzales, 2015; Neto et al., 2017; Ali et al., 2019) and its CWRs, *S. torvum* Swartz 1788 (turkey berry) (Ranil et al., 2015; Cutti and Kulckzynski, 2016; Ozden and Demit, 2016; Sarathkumar et al., 2017), *S. macrocarpon*, *S. aethiopicum*, and *S. incanum* (Gisbert et al., 2011). However, much more studies are still necessary to cover the huge biodiversity of this horticultural crop and its wild relatives. In this work, we focus on the CWR *S. villosum* Miller (hairy nightshade) which is consumed as leafy vegetable crop in Africa and it is part of the *S. nigrum* complex (African nightshade) including African indigenous vegetables with high nutritional content (Ojiewo et al., 2013), medicinal properties and secondary metabolites with potential insecticidal activity (Yuan et al., 2018). *S. villosum*, cultivated in Nyanza and Western Kenya, is appreciated for high adaptability, fast growth and easy seed production, however, low-quality seeds are currently used, thus affecting production (Gaya et al., 2007; Kimaru et al., 2019). The *S. villosum* seeds used in the present work were selected based on their fast germination profile, unique within a group of 14 eggplant CWR accessions available at CREA-GB in Montanaso Lombardo (Italy). The underpinning idea was to test to what extent priming would still be able to impact the pre-germinative metabolism, possibly highlighting some peculiar profile of specific components. To this purpose, an optimized hydropriming protocol was set and used to establish a working system for the study of the seed pre-germinative metabolism in this CWR, in order to find out changes in the seed antioxidant profile imposed by the treatment and select parameters that might be used in a larger screening of the available eggplant CWR accessions. Reactive Oxygen Species (ROS), lipid peroxidation and tocopherols, as well as polyphenols were investigated and results integrated to provide an original multilevel profile of the seed antioxidant environment.

## Materials and Methods

### Germination Tests and Hydropriming

**FIGURE 1:**
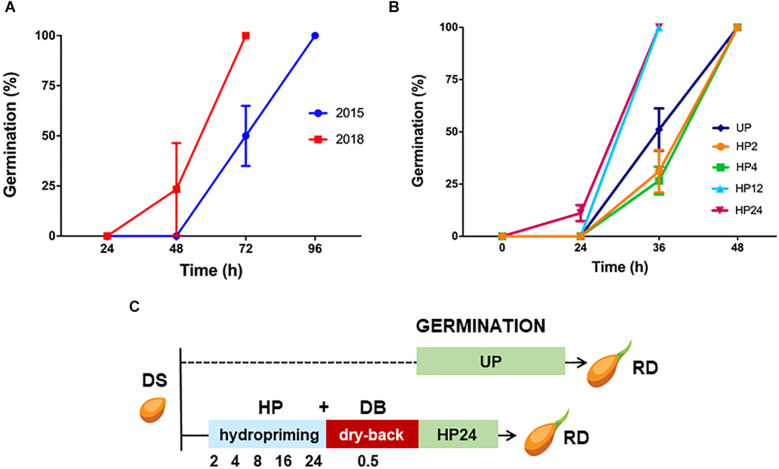
**(A)** Germination curve of the *S. villosum* seed lots collected in 2015 and 2018. Values are expressed as mean ± SD of three independent replicates with 15 seeds for each replicate. Statistical analysis was carried out using Two-way ANOVA (see Table 1B). **(B)** Results from germination tests carried out with *S. villosum* seeds (collected in 2015) and treated with hydropriming for 2, 4, 12, and 24 h and with unprimed (UP) seeds. Values are expressed as mean ± SD of three independent replications with 15 seeds for each replication. Statistical analysis was carried out using Two-way ANOVA (see Table 2B). **(C)** Experimental design for the study of the seed response to the hydropriming treatment HP24 in *S. villosum*. DS, dry seed. HP, hydropriming. DB, dry-back. UP, unprimed. RD, radicle protrusion.

*Solanum villosum* Miller (hairy nightshade) seeds of the accession CGN23849 (obtained from Center for Genetic Resources -Netherlands) were extracted from physiologically ripe fruits produced by plants cultivated in greenhouse at CREA-GB in Montanaso Lombardo (Italy). The seed lots hereby tested were collected in 2015 and 2018, respectively, each one consisting of seeds from approximately 100 fruits. Germination tests were performed as described (Forti et al., 2020a). Germination parameters are listed in Supplementary Table 1. For hydropriming, 45 seeds (15 seeds for each replicate) were soaked at 24°C for 2, 4, 12, and 24 h, hereby named HP2, HP4, HP12, and HP24) in 400 ml H_2_O under aeration produced by a Wave Air Pump Mouse 2 Beta aerator (De Jong Marinelife B.V., Netherlands) with the following parameters: 220–240 V, 50 Hz, 2.3 W, output 1.8 l min^–1^, pressure 0.012 MPa. At the end of the treatment, imbibed seeds were collected and transferred into glass tubes, placed between two cotton disks, covered with silica beads (disidry^®^ Orange Silica Gel, The Aerodyne, Florence, Italy) with a seed: silica ratio of 1:10 (w/w), and kept at 24–25°C for 0.5 h to reach the weight of dry seed. Such dehydration step is known as “dry-back”. For each hydropriming treatment and for the untreated control, seedlings were harvested at 7 days after the start of imbibition, and the fresh weight was measured. As for the selected hydropriming treatment HP24, the experimental design hereby used to study the seed response is shown in Figure 1C. Seeds were harvested at the indicated timepoints (2, 4, 8, 16, and 24 h of hydropriming, and after 0.5 h of dry-back). All samples we stored in in liquid N_2_ for molecular analyses.

### ROS Detection

The fluorogenic dye 2′,7′-dichlorofluorescein diacetate (DCFH-DA; Sigma-Aldrich, Milan, Italy) was used to quantify ROS levels as described (Macovei et al., 2016; Forti et al., 2020a). *S. villosum* seeds were collected (three seeds per time point), dried on filter paper and incubated for 15 min with 50 ml of 10 mM DCFH-DA. Fluorescence was determined at 517 nm using a Rotor-Gene 6000 PCR apparatus (Corbett Robotics, Brisbane, Australia), setting the program for one cycle of 30 s at 25°C. A sample containing only DCFH-DA was used to subtract the baseline fluorescence. Relative fluorescence was calculated by normalizing samples to controls and expressed as Relative Fluorescence Units (R.F.U.). The experiment was conducted in triplicate.

### MDA Determination

Malondialdehyde was quantified according to Sari et al. (2012) and Zeb and Ullah (2016), with the following modifications. Seeds were grounded to a fine powdery flour (granulometry from 20 to 200 mm)in a mixer mill type MM200 (Retch, Germany) for 30 s with the frequency set at 1/30. The resulting powder (200 mg) was mixed with 5 ml of H_2_O:0.5 M HClO_4_ solution (4:1) and few drops of 2% BHT (butylated hydroxytoluene, Sigma-Aldrich) in ethanol, to precipitate proteins. Samples were centrifuged (4°C, 10 min) and filtered with Whatman No. 1 paper (Whatman Limited, United Kingdom). MDA was determined as a thiobarbituric acid reactive substance (TBARS) following reaction with thiobarbituric acid (TBA) at high temperature. For each sample, an aliquot (100 μl) was mixed with 100 μl of TBA in 1 ml H_2_O and the mixture was heated at 95°C for 60 min. Samples were cooled at room temperature and absorbance measured at 254 nm using an UV-visible spectrophotometer (UV-1800, Shimadzu, United Kingdom). The standard MDA solution (Sigma-Aldrich; 100 μl, in a range of 0.025–0.1 mg ml^–1^) was added in a 1 ml test tube and mixed with TBA (100 μl), as previously described (Supplementary Figure 1A). Analyses were performed in triplicates.

### Analysis of Tocopherols and Phenolic Compounds

Extraction of tocopherols was performed as described (Kurilich and Juvik, 1999; Doria et al., 2009) with the following modifications. Seed powder (300 mg) was added to 5 ml of ethanol containing 0.1% butylated hydroxytoluene (BHT) and the mixture was incubated for 10 min at 85°C. Subsequently, samples were subjected to saponification by adding 150 μl of 80% KOH and incubating for 10 min. After adding 3 ml of H_2_O, samples were placed in ice bath for 3 min and 3 ml of pure hexane were added. After shaking for 10 min at 800 rpm and centrifuging at 12,000 rpm, the upper layer was transferred into a separate test tube, and the pellet (together with the remained layer of water) was re-extracted twice using 2 ml of hexane. The combined hexane fractions were washed with 3 ml of deionized H_2_O, vortexed, centrifuged for 10 min, and transferred into another test tube. Hexane fractions were dried using a vacuum evaporator and the residue dissolved in 200 μl acetonitrile:methanol:methylene chloride (45:20:35 v/v/v) prior to injection into the HPLC system (Kontron Instrument 420 system; Kontron Instruments, Munich, Germany) equipped with a C18 column (Zorbax ODS column 250 mm × 4.6 mm, 5 μ, Agilent Technologies). The isocratic mobile phase consisted of acetonitrile:methanol (60:40 v/v), the flow rate was 1.0 ml min^–1^ at room temperature, and absorbance was measured at 220 nm. As standard, γ-tocopherol (Sigma-Aldrich) was used for a calibration curve (Supplementary Figure 1B). Polyphenol content was assayed as described (Sadiq et al., 2014) by an HPLC system (Kontron Instrument 420 system) (Kontron Instruments, Munich, Germany) equipped with a reverse phase C18 column (SepaChrom^®^ —Robusta, 100 A, 5 μ, 250 mm × 4.6 mm) and UV detector. The mobile phase, fluxed at a rate of 0.8 ml min^–1^, consisted of 4% acetic acid (solvent A) and pure methanol (solvent B) according to the gradient shown in Supplementary Table 2.

### Statistical Analysis

The effects of priming versus unprimed control in terms of germination percentage, days, and their interaction were analyzed using Two-way ANOVA (Analysis of Variance) (^∗^*P* < 0.05; ^∗∗^*P* < 0.01; ^∗∗∗^*P* < 0.001; ^****^P < 0.0001) carried out with the statistical software GraphPad Prism 8 (GraphPad Software Inc., San Diego, CA, United States). Comparison between unprimed control and different priming treatments were carried out using the *post hoc* Tukey’s HSD (Honest Significant Difference) test. Means with a significance value lower than 0.05 (*P* ≤ 0.05) were considered statistically different. Statistical analysis of phenotyping data, MDA, tocopherols, and polyphenols data was performed using the Student’s *t*-test (^∗^*P* < 0.05; ^∗∗^*P* < 0.01; ^∗∗∗^*P* < 0.001).

## Results

### Impact of Hydropriming on Fast Germinating *Solanum villosum* Seeds

Two *S. villosum* seed lots collected in 2015 and 2018, respectively, were first analyzed to assess germination performance. Each lot included seeds from approximately 100 fruits (15–20 seeds per fruit, as reported by Haque et al., 2018) covering the genetic variability within this CWR. As shown in Figure 1A, the seed lot of year 2015 started germination between the 2nd and 3rd day following imbibition, reaching 100% at the 4th day. Germination of seeds collected in 2018 started earlier that the seed lot of year 2015 between the 1st and 2nd day following imbibition, reaching 100% at the 3rd day. Thus, both the *S. villosum* seed lots were characterized by a fast germination profile, compared to domesticated eggplant seeds previously characterized (Forti et al., 2020a). The detailed list of germination parameters shown in Table 1A reflects the observed differences between seed lots, e.g., the *CVG* (coefficient of velocity of germination) value increased from 521.42 ± 25.26% (2015) up to 875.01 ± 23.22% (2018). Statistical analysis of data is shown in Table 1B.

**TABLE 1:** **(A)** Germination parameters calculated based on results of germination tests carried out on the *S. villosum* seed lots collected in 2015 and 2018, respectively. **(B)** Results of Two-way ANOVA analysis comparing germination parameters of the *S. villosum* seed lots collected in 2015 and 2018, respectively, carried out using the *post hoc* Tukey’s HSD test. Values are expressed as means ± SD from three independent replicates. Asterisks indicate statistically significant differences between the two seed lots determined using Student’s *t* test (*P* < 0.05). G, germinability; MGT, mean germination time; CVG, coefficient of velocity of germination; MGR, mean germination rate; U, uncertainty; Z, synchronization index. *P < 0.05, **P < 0.01, ***P < 0.001, and ****P < 0.0001.

**(A)**						
**Seed lot**	***G* (%)**	***MGT* (days)**	***CVG* (%)**	***MGR* (day^–1^)**	***U* (bit)**	***Z* (unit less)**

2015	100.0 ± 0.00	2.49 ± 0.10	521.42 ± 25.26	5.0 × 10^–3^ ± 9.2 × 10^–5^	0.98 ± 0.02	0.48 ± 0.01
2018	100.0 ± 0.00	2.10 ± 0.05	875.01 ± 23.22****	1.2 × 10^–3^ ± 15.0 × 10^–5^ ***	0.46 ± 0.16**	0.81 ± 0.01**
**(B)**						

**Comparison**	***q***	**DF**	***P* value**	**Significance**		

2015–2018	204.6	4	0.0001	****		

Based on the reported data, the oldest seed lot (year 2015) was selected for hydropriming treatment. Although such an accelerated germination phenotype would not apparently require further improvement, we wanted to test whether priming would be able to impact the pre-germinative metabolism of *S. villosum* seeds, eventually disclosing significant changes and/or strengthening the role of specific antioxidant components. Results from germination tests performed with unprimed (UP) seeds and seeds treated for increasing time are shown in Figure 1B whereas germination parameters are listed in Table 2A. *S. villosum* seeds were soaked in H_2_O for 2, 4, 12, and 24 h (hereby named hydropriming treatments HP2, HP4, HP12, and HP24), subjected to dry-back (DB, 0.5 h) and then used for germination tests. According to germination parameters reported in Table 2A, the HP12 and HP24 treatments were able to accelerate germination. The *MGT* (mean germination time) values decreased from 2.49 ± 0.10 days (UP) to 2.00 ± 0.00 days (HP12) and 1.89 ± 0.04 days (HP24). The positive impact of hydropriming on the *S. villosum* seeds was also evident when considering the *CVG* (coefficient of velocity of germination) value, that increased from 521.42 ± 25.26% (UP) up to 672.81 ± 0.00% (HP12) and 718.91 ± 16.63% (HP24). Statistical analysis of data is shown in Table 2B. In order to assess the impact of hydropriming treatments at the seedling level, biometric parameters were measured on *S. villosum* seedlings collected 7 days following imbibition. Results are shown in Table 3. Statistical analysis highlighted significant changes in root length of seedlings resulting from the HP24 treatment, compared to those from UP seeds. Based on the germination profile and phenotyping analysis, the HP24 hydropriming protocol was selected for subsequent molecular analyses.

**TABLE 2:** **(A)** Germination parameters calculated based on results of germination tests carried out on *S. villosum* seeds (seed lot collected in 2018) treated with hydropriming (HP) for increasing time (2 h, 4 h, 12 h, and 24 h) and unprimed (UP) seeds. **(B)** Results of Two-way ANOVA analysis comparing germination parameters of the unprimed (UP) *S. villosum* seeds versus primed seeds (HP2, HP4, HP12, and HP24 treatments) carried out using the *post hoc* Tukey’s HSD test (*P* ≤ 0.05). Values are expressed as means ± SD from three independent replicates. Asterisks indicate statistically significant differences between primed and unprimed seeds determined using Student’s *t* test (*P* < 0.05). *G*, Germinability. *MGT*, Mean germination time. *CVG*, Coefficient of velocity of germination. *MGR*, Mean germination rate. *U*, Uncertainty. *Z*, Synchronization index. *P < 0.05, **P < 0.01, ***P < 0.001, and ****P < 0.0001.

**(A)**						
**Treatment**	***G* (%)**	***MGT* (days)**	***CVG* (%)**	***MGR* (day^–1^)**	***U* (bit)**	***Z* (unit less)**

UP	100.0 ± 0.00	2.49 ± 0.10	521.42 ± 25.26	5.0 × 10^–3^ ± 9.2 × 10^–5^	0.98 ± 0.02	0.48 ± 0.01
HP2	100.0 ± 0.00	2.69 ± 0.10	474.79 ± 21.72	0.021 ± 9.8 × 10^–5^	0.87 ± 0.13	0.56 ± 0.09
HP4	100.0 ± 0.00	2.73 ± 0.07*	465.06 ± 13.92*	2.2 × 10^–3^ ± 6.4 × 10^–5^	0.83 ± 0.1	0.58 ± 0.07
HP12	100.0 ± 0.00	2.00 ± 0.00*	672.81 ± 0.00**	1.5 × 10^–3^ ± 0.00*	0.00 ± 0.00***	1.00 ± 0.00***
HP24	100.0 ± 0.00	1.89 ± 0.04**	718.91 ± 16.63*	1.4 × 10^–3^ ± 3.38 × 10^–5^**	0.50 ± 0.12 *	0.78 ± 0.07*
**(B)**						

**Comparison**	**q**	**DF**	***P* value**	**Significance**		

UP vs HP2	4.744	10	0.0453	*		
UP vs HP4	5.798	10	0.0144	*		
UP vs HP12	11.6	10	<0.0001	****		
UP vs HP24	14.23	10	<0.0001	****		

**TABLE 3:** Results of phenotyping analyses performed on *S. villosum* seven-day old seedlings developed from seeds treated with hydropriming (HP2, HP4, HP12, and HP24) and unprimed (UP) seeds.

**Parameter**	**UP**	**HP2**	**HP4**	**HP12**	**HP24**
Fresh weight (mg/15 seedlings)	13.20 ± 0.002	13.70 ± 0.003	12.20 ± 0.0007	14.00 ± 0.002	9.30 ± 0.002*
Dry weight (mg/15 seedlings)	0.80 ± 0.003	1.90 ± 0.001	0.90 ± 0.0002	1.10 ± 0.0002	0.90 ± 0.0002
Radicle length (mm)	62.00 ± 2.870	64.00 ± 4.36	66.00 ± 2.33	74.00 ± 1.23*	69.00 ± 5.87*

Values are expressed as mean ± SD of three independent replications with 15 seedlings for each replication. Asterisks indicate statistically significant differences determined using Student’s *t*-test. *P < 0.05.

### Hydropriming on Fast Germinating *Solanum villosum* Seeds Enhances Tocopherols Accumulation

**FIGURE 2:**
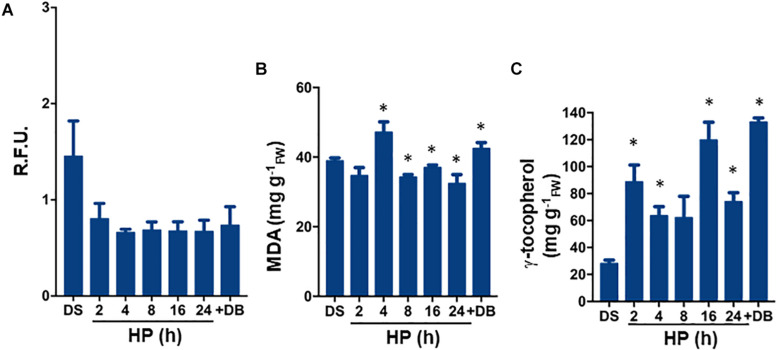
**(A)** ROS levels measured during hydropriming as well as at the end of dry-back, using the DCF-DA fluorescent dye. **(B)** Quantification of lipid peroxidation levels by MDA assay carried out during hydropriming as well as at the end of dry-back. **(C)** Total tocopherol content assessed during hydropriming as well as at the end of dry-back. Values are expressed as mean ± SD of three independent replicates with 15 seeds for each replicate. Asterisks indicate statistically significant differences determined using Student’s *t*-test (*P* < 0.01). DB, dry-back; DCF-DA, dye 2′,7′-dichlorofluorescein diacetate; DS, dry seed; FW, fresh weight; HP, hydropriming; MDA, malondialdehyde; R.F.U., relative fluorescence unit; ROS, reactive oxygen species.

The experimental design set for *S. villosum* seeds is shown in Figure 1C. No significant changes (*P* = 0.08) in ROS levels were observed at 2 h of treatment (0.80 ± 0.16 R.F.U.), compared to DS (dry seed, 1.45 ± 0.37 R.F.U.). ROS were maintained at similar levels at the subsequent timepoints until the dry-back was completed (Figure 2A). In order to evaluate the level of oxidative damage, lipid peroxidation was assessed by measuring malondialdehyde (MDA) (Figure 2B). The estimated amount of MDA in the DS was 38.88 ± 0.88 μg/g_*FW*_ whereas no significant (*P* = 0.07) changes were detected after 2 h of treatment. At the subsequent timepoints, and then following dry-back, the estimated MDA levels were still significantly lower than DS (Figure 2B). Thus, the *S. villosum* seeds did not undergo relevant changes in lipid peroxidation along hydropriming, possibly due to enhanced antioxidant response. To investigate this aspect, tocopherols were also measured. The estimated tocopherol content in *S. villosum* dry seeds was 28.00 ± 2.60 mg/g_*FW*_ (Figure 2C). After 2 h of treatment, tocopherol levels were significantly (*P* = 0.04) increased (88.60 ± 12.50 μg/g_*FW*_). The highest levels (133.00 ± 0.00 μg/g_*FW*_) were recorded after dry-back (*P* = 0.046) (Figure 2C). Based on these results, both accumulation of tocopherols triggered during the hydropriming treatment as well as limited lipid peroxidation can be envisaged as beneficial effects associated with this treatment.

### Hydropriming Applied to *Solanum villosum* Seeds Resulted in the Accumulation of Phenolic Compounds

**FIGURE 3:**
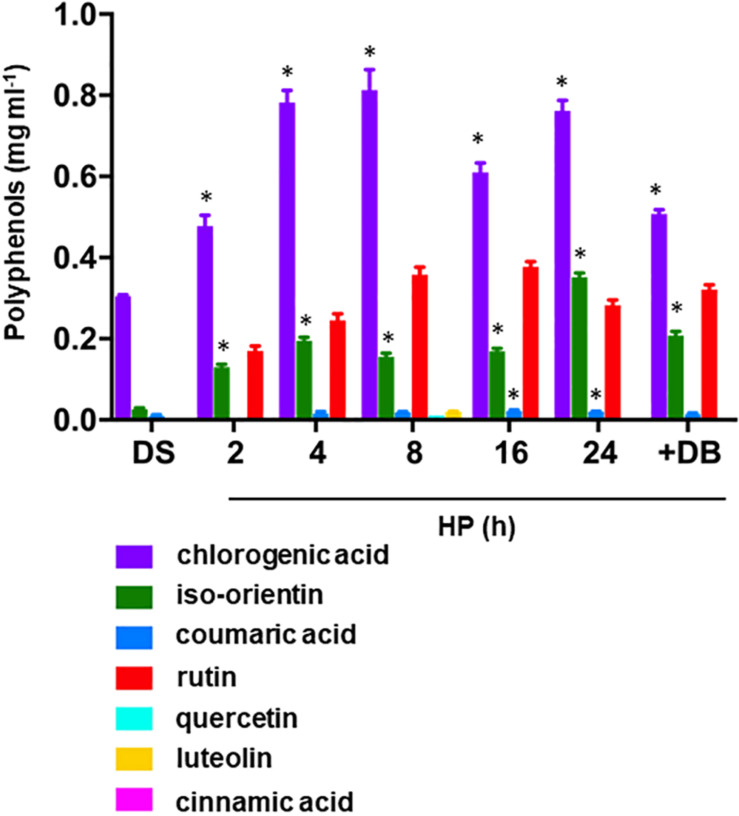
Accumulation of phenolic compounds occurring in *S. villosum* seeds during hydropriming as well as at the end of dry-back. Values are expressed as mean ± SD of three independent replicates with 15 seeds for each replicate. Asterisks indicate statistically significant differences determined using Student’s *t*-test (*P* < 0.01). DB, dry-back; DS, dry seed; FW, fresh weight; HP, hydropriming; RD, radicle protrusion; UP, unprimed.

The profiles of phenolics in *S. villosum* seeds subjected to hydropriming are shown in Figure 3. In the dry seed, chlorogenic acid [(1*S*,3*R*,4*R*,5*R*)-3-{[(2*E*)-3-(3,4-dihydroxyphenyl)prop-2-enoyl]oxy}-1,4,5-trihydroxycyclohexanecarboxylic acid], an ester of caffeic acid and (-)-quinic acid) was the main component detected (0.305 mg ml^–1^), followed by iso-orientin [2-(3,4-dihydroxyphenyl)-5,7-dihydroxy-6-[(2S,3R,4R,5S,6R)-3,4,5-trihydroxy-6-(hydroxymethyl)oxan-2-yl]chromen-4-one], a 6-C-glucoside of luteolin (0.023 mg ml^–1^), and coumaric acid [(2*E*)-3-(4-Hydroxyphenyl)prop-2-enoic acid], a phenolic derivative of cinnamic acid (0.012 mg ml^–1^) (Figure 3, DS). Hydropriming stimulated accumulation of chlorogenic acid in *S. villosum* seeds at 2 h, with maximum levels at 4–8 h (0.804 and 0.777 mg ml^–1^, respectively). The flavone iso-orientin increased significantly (*P* = 0.01) at 2 h of hydropriming (0.134 mg ml^–1^, 5.8-fold, compared to DS) (Figure 3). The level of coumaric acid was not significantly affected by hydropriming whereas *ex novo* accumulation of rutin {2-(3,4-dihydroxyphenyl)-5,7-dihydroxy-3-[α-L-rhamnopyranosyl-(1→6)-β-D-glucopyranosyloxy]-4*H*-chromen-4-one} occurred at 2 h (0.169 mg ml^–1^) and significantly increased during the treatment (up to 0.345 mg ml^–1^; 8 h) (Figure 3). Hydropriming also induced *ex novo* accumulation of other phenolic compounds, although at very low levels, namely quercetin [2-(3,4-dihydroxyphenyl)-3,5,7-trihydroxy-4*H*-chromen-4-one] and luteolin [2-(3,4-dihydroxyphenyl)-5,7-dihydroxy-4-chromenone] (0.01 and 0.02 mg ml^–1^, respectively; 8 h) (Figure 3). It is worth noting that the phenolic compounds chlorogenic acid and iso-orientin were significantly enriched following dry-back, whereas rutin appeared as a novel component, compared to DS. At the moment, we can only speculate about the possible role of these compounds in the protection of *S. villosum* seeds against desiccation-associated oxidative damage.

## Discussion

In this work, we explore for the first time the seed response to hydropriming in the eggplant CWR *S. villosum*. A preliminary screening of germination profiles, carried out with unprimed seeds from two different lots, revealed that the process was faster in *S. villosum*, compared to other CWRs available at the CREA-GB germplasm collection. The current literature on *S. villosum* is still scanty, particularly as concerns seed traits. It has been reported that *S. villosum* seed can germinate within 7 days and seedlings show fast growth (Fern, 2021). Although priming usually targets low-quality seeds, we were wondering whether stimulation of the pre-germinative metabolism could be used as another strategy to disclose changes in the tested parameters and indicators.

Hydropriming applied to the fast germinating *S. villosum* seeds (namely the HP24 treatment, adapted from a previous protocol designed for the *S. melongena* inbred line “67/3” by Forti et al., 2020a), was associated with limited lipid peroxidation and significant tocopherols enhancement, suggesting that the treatment provides protection against membrane oxidative damage, in line with the observations made in other plant species (Zhang et al., 2007; Zheng et al., 2015). To date, there are studies describing the profiles of bioactive compounds, including g and a tocopherols in *S. villosum* leaves extract (Venkatesh et al., 2014), shoots and roots (Okello et al., 2017), fresh and dry stems and leaves (Louh et al., 2014) but, to our knowledge, this is the first investigation describing the profiles of tocopherols in *S. villosum* seeds.

Accumulation of phenolic compounds triggered by seed priming has been reported for other species (Farooq et al., 2017). It is possible that the observed polyphenols profiles might provide beneficial effects also in the eggplant CWR. These secondary plant metabolites, synthesized during growth and reproduction as well as in response to biotic and abiotic stress, contribute to the nutritional value of eggplant and its CWRs, and particularly has made possible to classify eggplant as a functional food (Niño-Medina et al., 2017). Chlorogenic acid, known for its beneficial properties, is currently the target of eggplant breeders for the development of new cultivars with increased content (Plazas et al., 2013). The flavone iso-orientin shows anti-cancer activities whereas rutin is a flavonoid with antiallergic, anti-inflammatory, and antiproliferative activities. Data hereby reported show that hydropriming impacts the phenolics profile of this CWR, enhancing accumulation and even promoting *ex novo* synthesis of specific compounds. As previously discussed for tocopherols, the occurrence of polyphenols in *S. villosum* has been so far assessed only in tissues other than seeds, such as in seedlings challenged with salinity stress (Ben-Abdallah et al., 2019), and leaf extracts (Venkatesh et al., 2014; Yuan et al., 2018).

It is generally acknowledged that the seed response to priming is strictly genotype-dependent and this divergence is expressed through significant changes of metabolic indicators (Carrillo-Reche et al., 2018). In compliance with this observation, Forti et al. (2020a) demonstrated that hydropriming carried out for 24 h in the eggplant line “67/3” caused a significant increase in ROS amounts. The oxidative window model states germination release by ROS through protein oxidation (Bailly et al., 2008), as a result of a delicate balance between the free radicals amount and cellular antioxidant systems. It is also worth noting that seed lots of another eggplant CWR, *S. torvum* Swartz 1788 revealed decreased ROS levels when treated with hydropriming (Forti, 2020). It could be hypothesized that the different responses occurring during hydropriming might reflect different ROS contents in the dry seed, determined by maternal and environmental factors (Sano et al., 2016). Deciphering the biological bases of hydropriming is still an open question. Soaking seeds with water might in some way resemble the environmental condition of flooding stress. Indeed, it has been reported that hydropriming can improve the ability of rice seeds to withstand the deleterious effects of flooding, by shortening the emergence time (Mulbah and Adjetey, 2018; Sarkar, 2020). Flooding-tolerant seeds are also desirable products for breeding in legumes (Sayama et al., 2009). Among Solanaceae, this is an appreciated trait in eggplant germplasm collections (Bhatt et al., 2014) as Latifah et al. (2019) showed that tomato plants grafted on eggplant rootstocks display enhanced water logging tolerance. However, the correlation between flooding stress tolerance and hydropriming has still to be established in terms of physiological and molecular mechanisms.

The timing and conditions of the dry-back step are crucial for a successful priming protocol (Chen and Arora, 2011). No significant impact on ROS content was observed in the primed *S. villosum* seeds subjected to dehydration, differently from what reported for the “67/3” line and *S. torvum* (Forti et al., 2020a; Forti, 2020). This puzzling scenario is evident in other case studies, e.g., Kubala et al. (2015) found decreased ROS levels in rape (*Brassica napus* L.) primed seeds during dry-back. Taken together, this information suggests that the treatment impacts the redox environment, bringing the seed toward a genotype-specific set of the pre-germinative metabolism compatible with improved germination performance.

The impressive biodiversity of eggplant CWRs brings novel opportunities and challenges to plant scientists and breeders in their race against time to find out sustainable strategies for fighting global climate change. This work explores for the first time the molecular dynamics of the pre-germinative metabolism in the CWR *S. villosum* that is part of a huge germplasm collection currently under evaluation for breeding purposes. The molecular profile of the response to priming, hereby provided for *S. villosum*, highlights the inherent complexity of this issue. Translating such knowledge to other CWRs might be a difficult task, however, there are some promising hints that deserve further investigation. The successful hydropriming treatment will be used to shape germination in other accessions of the germplasm collection for research purposes and/or made available to horticultural breeders specialized in *Solanaceae.* Indicators (ROS, tocopherols, phenolics) will be tested in a more representative set of different CWR genotypes to figure out a reliability threshold for their use as predictive hallmarks of seed quality.

## Data Availability Statement

The original contributions presented in the study are included in the article/Supplementary Material, further inquiries can be directed to the corresponding author/s.

## Author Contributions

AB, CF, LB, LT, and GLR conceived and designed the study. CF, ED, LB, LT, VO, and AP performed the experiments. AB, CF, and LB wrote the manuscript. LT, GLR, AM, AP, and ED reviewed the manuscript. All authors read and approved the final manuscript.

## Conflict of Interest

The authors declare that the research was conducted in the absence of any commercial or financial relationships that could be construed as a potential conflict of interest.
