# Supplementary material for: Hydropriming Applied on Fast Germinating Solanum villosum Miller Seeds: Impact on Pre-germinative Metabolism
Source: Front Plant Sci. 2021 Mar 25;12:639336. doi: 10.3389/fpls.2021.639336 (PMC8030258; doi:10.3389/fpls.2021.639336)
Supplement: Supplementary file 1 [file Data_Sheet_1.PDF]

Supplementary Material

Supplemental Data

Supplementary Table S1. Germination parameters used in this study to assess the efficacy of hydropriming on *S. villosum* Miller seeds as reported by Ranal and Garcia de Santana (2006). For each parameter, definition, formula, limits of measurement and unit are shown.

Parameter	Formula	Limits	Unit
G<br>mean number of germinated seeds per day expressed in percentage	*G* = (100 x n. of germinated seeds)/ Total n. of seeds	$0 \leq G \leq 100$	%
MGT<br>mean germination time (*)	$\bar{t} = \sum_{i=1}^{k} n_i t_i / \sum_{i=1}^{k} n_i$	$0 < t \leq k$	day
CVG<br>coefficient of velocity of germination	$CVG = (\sum_{i=1}^{k} f_i / \sum_{i=1}^{k} f_i x_i)100$	$0 < CVG \leq 100$	%
MGR<br>mean germination rate	$v = CV/100$	$0 < v \leq 1$	day^{-1}
U<br>uncertainty associated to the distribution of the relative frequency of germination (**)	$\overline{E} = -\sum_{i=1}^{k} f_i \log_2 f_i$, being $f_i = n_i / \sum_{i=1}^{k} n_i$	$0 \leq U \leq \log_2 n$	bit
Z<br>synchronization index (***)	$Z = \sum C_{n_i,2} / N$	$0 \leq Z \leq 1$	unitless

(*) t_i is time from the start of the experiment to the ith observation (day); n_i: number of seeds germinated in the time *i* (not the accumulated number, but the number correspondent to the ith observation), and *k* is the last time of germination.
(**) f_i is the relative frequency of germination, n_i the number of seeds germinated on the day *i*, and *k* the last day of observation
(***) $C_{ni,\ 2}$: combination of the seeds germinated in the time *i*, two together, and n_i the number of seeds germinated in the time *i*.

Reference

Ranal, M.A., and Garcia de Santana, D. (2006). How and why to measure the germination process? *Brazilian J. Bot.* 29, 1-11. doi: 10.1590/S0100-84042006000100002

A

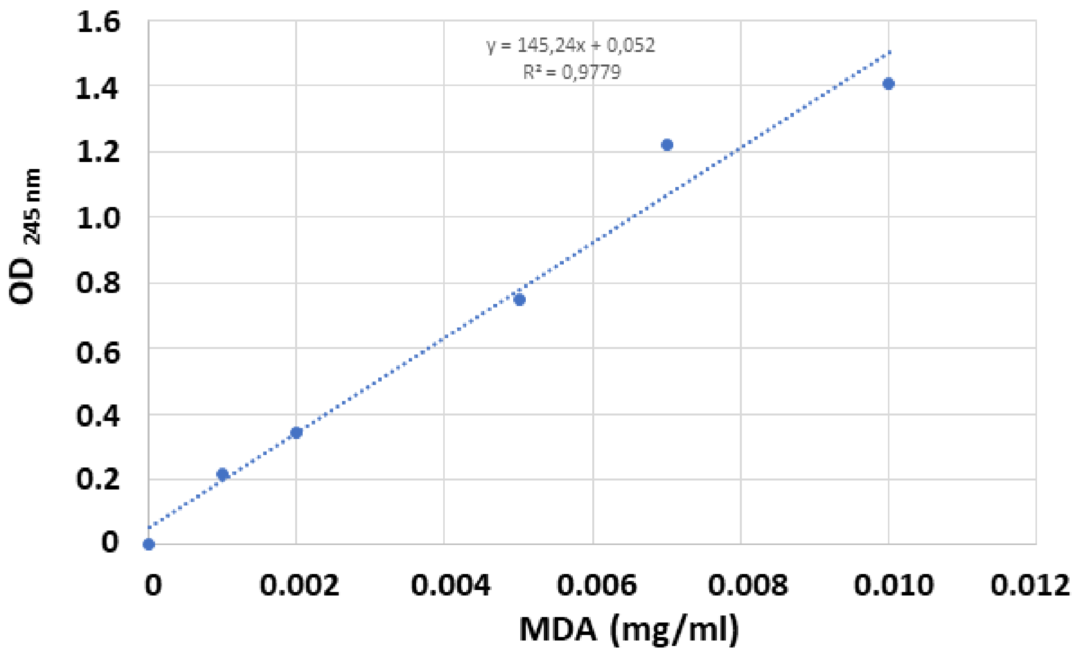

B

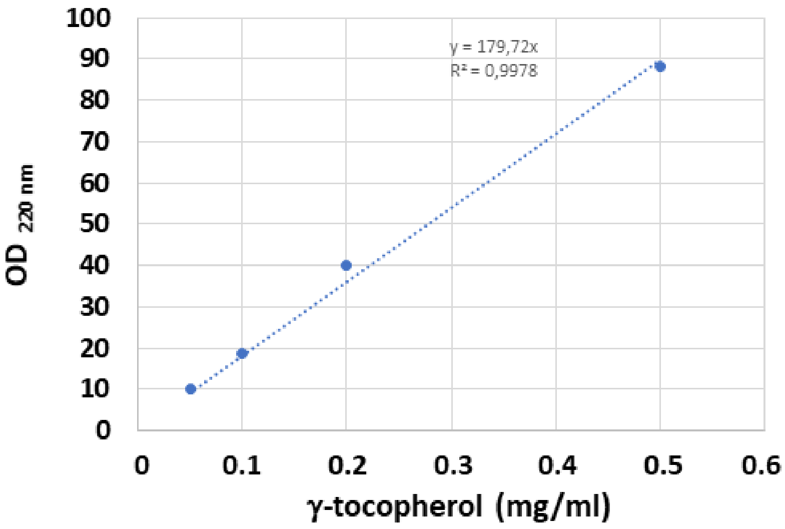

Supplementary Fig. S1. (A) MDA standard curve measured at 254 nm using an UV-visible spectrophotometer (UV-1800, Shimadzu, U.K.). **(B)** Standard curve for γ-tocopherol measured at 220 nm using an UV-visible spectrophotometer (UV-1800, Shimadzu, U.K.).

Supplementary Table S2. HPLC gradient of the mobile phase, used for polyphenols determination.

Minutes	% A (4% acetic acid)	% B (100% methanol)
1-4	100	0
4-10	100	0
10-22	50	50
22-24	10	90
24-26	50	50
26-27	90	10
